# Discounting money and health effects from communicable and noncommunicable diseases in Thailand

**DOI:** 10.1038/s41598-023-30559-2

**Published:** 2023-02-27

**Authors:** Jirawit Yadee, Unchalee Permsuwan, Kansinee Guntawongwan, Woraluck Himakalasa, Piyaluk Buddhawongsa

**Affiliations:** 1 Degree Program in Pharmacy, Faculty of Pharmacy, Chiang Mai University, Chiang Mai, Thailand; 2 Department of Pharmaceutical Care, Faculty of Pharmacy, Chiang Mai University, Chiang Mai, Thailand; 3 Center for Medical and Health Technology Assessment (CM-HTA), Department of Pharmaceutical Care, Faculty of Pharmacy, Chiang Mai University, Chiang Mai, Thailand; 4 Center of Human Resource and Public Health Economics (CHPE), Faculty of Economics, Chiang Mai University, Chiang Mai, Thailand

**Keywords:** Health care, Medical research

## Abstract

The purpose of this study was to determine the discount rates for money and health outcomes in the Thai context, including the discount rates for communicable and non-communicable diseases. Moreover, this study aimed to explore the socio-demographic characteristics that influence discounting. The computer-based experimental design was used to obtain time preferences for money and health in a total of 1202 Chiang Mai province population, aged 25–50, individually interviewed by trained interviewers. Money-related questions were carried out in all subjects. For health-related questions, all subjects were randomly assigned in a 1:1 ratio for response to questions about Coronavirus Disease 2019 (COVID-19) (N = 602) and air pollution (N = 600). A choice-based elicitation procedure was performed in the experiment to obtain the indifference values from subjects’ time preferences. The cumulative weighting functions were generated using the indifference values to indicate the degree of discounting. The discount factors were computed from the cumulative weighting functions. The discount rates were estimated using a continuous approximation based on the relationship between the discount factors and the parameters governing the discounting model. The Tobit model was applied to investigate the relationships between discounting and socio-demographic characteristics. Discounting for money was greater than discounting for health. Money and health had annual discount rates of 6.2% and 1.3%, respectively. Furthermore, in the COVID -19 situation, the annual discount rate for health was higher than that in the air pollution situation (2.4% vs. 0.7%). Generation X subjects (aged 42 years and above), children under the age of 15 in the household, and underlying diseases were positively related to discounting, while household income was negatively related to discounting. Health should be discounted at a lower rate than money. Moreover, different discount rates should be considered for different types of diseases.

## Introduction

Discounting is a method of adjusting the future costs and benefits of healthcare interventions such as devices, medicines, vaccines, procedures, and healthcare systems to their present value. The impact of discounting, according to the context of economic evaluation, is dependent on the timing of costs and benefits, implying that society values future costs and benefits less than current costs and benefits. As a result, discounting costs and health outcomes should be taken into account^1^.

Several controversies in terms of theoretical rationale exist regarding whether costs and health benefits should be discounted at the same rate^2,3^. This is because health is unlike other resources. Hence, there is no direct opportunity to invest in it to produce future gains. Several arguments support using the same discount rate for costs and health outcomes. To begin, the consistency argument^4^ stated that future health outcomes are valued relative to costs, and thus future health outcomes should be discounted relative to current costs. Next, the paralyzing paradox^5^ revealed that new interventions with lower cost-effectiveness ratios are preferred to those with higher cost-effectiveness ratios when there is no or a lower discount rate for health outcomes than for costs. As a result, if the decision is postponed, the new interventions appear to be more cost-effective. However, some arguments exist to support using the different discount rates for costs and health outcomes. For instance, criticisms of the consistency argument^6^ argued that health benefits may change over time as technology improves, resulting in a lower cost to save lives, or it may become more expensive to save lives due to environmental or other factors. As a result, different discount rates for costs and health benefits should be considered. Furthermore, criticisms of the paralyzing paradox^7^ argued that the option of infinitely deferring health intervention is irrelevant to policymaking because budgets must be spent. Furthermore, the political nature of public decisions regarding resource allocation cannot be neglected. Therefore, the paradox has limited application in the real world.

Delay discounting is another issue that plays an important role in different outcomes. Based on the review study conducted by Odum et al.^8^, which finally included 53 articles for data analysis, it was found that: (1) in comparison to monetary outcomes, non-monetary outcomes were discounted more steeply. This result demonstrates that there is widespread and consistent evidence of steeper discounting of non-monetary outcomes across various outcomes, populations, and methodologies. (2) A positive correlation existed between the degree of delay discounting for monetary and non-monetary outcomes. This finding indicates that individuals who have a tendency to discount one outcome steeply also tend to discount other outcomes steeply.

Most of the recommendations for discounting costs and health outcomes are taken into action by government agencies or regulatory bodies. For example, the National Institute of Clinical Excellence (NICE), an organization in the United Kingdom (UK) that provides national guidance and advice to improve public health policy and social care, recommends discounting the costs and benefits of healthcare interventions at the same rate (3.5% per year)^9^. In addition, according to the Thai Guidelines for Health Technology Assessment (HTA), the recommended annual discount rate for the cost and outcome of the base-case analysis is 3%, and sensitivity analysis should be performed by varying the discount rate from 0 to 6%^10^. However, discounting at different rates is recommended in several countries. For instance, the National Health Care Institute of the Netherlands (Zorginstituut Nederland) recommends that the costs be discounted at a higher rate than the health outcomes, probably with the annual discount rate for costs and outcomes at 4% and 1.5%, respectively^11^. Moreover, the Belgian Health Care Knowledge Centre, an organization under the Ministry of Public Health and Social Affairs, Kingdom of Belgium, recommends that costs should be discounted at a rate of 3% and health effects at a rate of 1.5% annually^12^.

Previous studies on discounting in economic evaluation revealed that the choice experiment was commonly conducted to estimate the discount rate of monetary and/or health outcomes. The discount rate for monetary and health outcomes was found in the range of 2–28.5% and 3–29.4%, respectively^13–18^. Moore and Viscusi^13^ estimated the discount rates for health benefits by asking workers in their choices of job risk and wage to consider their future utility. The discount rate equaled approximately 2%^13^. Cairns^14^ conducted an empirical study to evaluate the discount rate in the UK. The mean financial discount rate was significantly higher than the health discount rate derived from the choices in terms of postponing or expediting a period of illness (28.5% vs. 3%)^14^. Cropper et al.^15^ found the median discount rates range from 17% for a five-year horizon to 3.7% for a horizon of 100 years. In addition, van der Pol and Cairns^16^ revealed the median rates of discount to be in the range of 5.5–9.1% for own health by measuring the time-preference rate between two scenarios occurred in terms of the days of current and future ill-health. A study by Attema et al.^17^ applied the direct method, which requires no assumptions about the utility function, to estimate the discount rate and found the median discount rates to be 6.5% for money and 2.2% for health outcomes. Fredslund et al.^18^ reported the estimated annual discount rates for money and health outcomes at 27.5% and 29.4%, respectively. These previous studies revealed controversies over discount rates based on the experimental design whether costs and health benefits should be discounted at the same rate.

In Thailand, the annual discount rates for cost and outcome at the same rate of 3% are still recommended for the economic evaluation of healthcare interventions in accordance with the Thai HTA guidelines^10^. However, these recommended discount rates may not be suitable or reflect Thailand’s current economic conditions. Furthermore, different risk perceptions can lead to risk-taking behaviors^19^, implying that the health consequences of communicable diseases may differ from those of non-communicable diseases. As a result, there are research gaps in investigating the appropriate discount rate for costs and health outcomes in Thailand, as well as whether the discount rates for communicable and non-communicable diseases should be the same. However, the limited transportation and travel measures imposed to control the spread of COVID-19 in Thailand during the time of this study entitled Chiang Mai Province in northern Thailand to be selected as the only representative area for the investigation.

Therefore, the purpose of this study was to determine the discount rates for costs and health outcomes in the Thai context, including discount rates for health derived from communicable and non-communicable diseases. Moreover, this study aimed to explore the socio-demographic characteristics that influence discounting.

## Methods

A computer-based experimental design was used, along with a choice-based elicitation procedure, to obtain time preferences for money and health. The experiment based on money-related questions was carried out in all subjects. For health-related questions, the subjects were divided into two subgroups, one to answer questions about COVID-19 and the other about air pollution caused by fine particulate matter. The data were collected from a large representative sample of the Chiang Mai Province population aged 25–50 years.

### Experimental design

An experimental design method from a previous study by Attema et al.^17^ was applied in this study. In brief, all subjects in the experiment were asked to choose between their money and health over the next 20 years based on their current age. There were two sets of questions (health- and money-related questions). The order of the question sets was randomly selected. Furthermore, the COVID-19 situation or respiratory diseases caused by air pollution respondents were also chosen at random for the health-related questions. Each health- or money-related question was completed by the subjects. The experiment was then run until finished or until the maximum number of iterations was reached, which was 4.

For each iteration, a choice-based elicitation procedure was performed to obtain the subjects’ time preferences. After subjects had chosen their preferred option, the next iteration was altered to make the chosen option less attractive and the non-chosen option more attractive. The indifference values were determined after the experiment was completed. Detailed information on eliciting indifference values is provided in the data analysis section.

**Figure 1:**
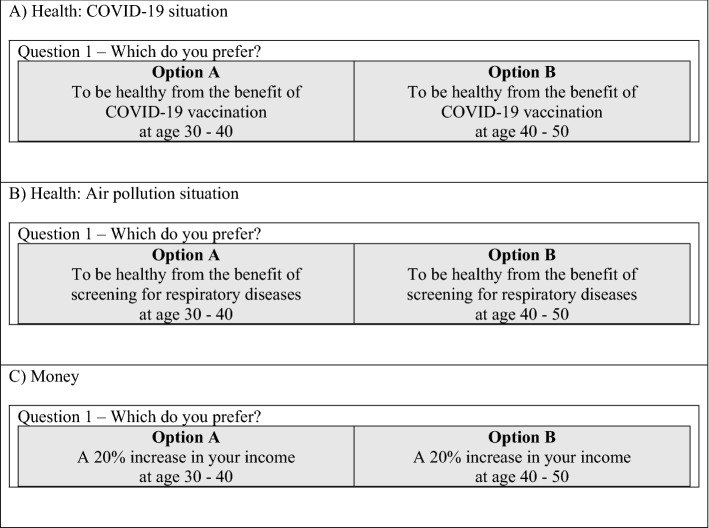
Example of questions about health and money. COVID-19, Coronavirus disease 2019.

In terms of health, the subjects were required to imagine that they had suffered from either COVID-19 or an air pollution situation. The interviewer informed the subjects that new health technology such as a vaccine or screening program was available and effective for disease treatment, resulting in the subjects’ full health state. The subjects were then asked to choose the most preferable period based on changes in their health condition (Fig. 1A and B). For the money-related questions, the subjects were asked to imagine that their income or purchasing power had increased by 20%. The interviewer asked the subjects to select their preferred period based on changes in their income (Fig. 1C).

### Population

The sample size was calculated using a formula by Vaughan et al.^20,21^ and adjusted by a design effect, which was a correction factor used to adjust the required sample size for cluster or complex sampling methods^22^. The modified formula was shown as follow.

$$N={\left[\frac{{Z}_{\alpha /2}V}{\Delta }\right]}^{2}D$$

N is the required sample size. Z_α/2_ is the percentage of (1−α) confidence interval statistic and refers to the critical value from the Z-score that corresponds to α/2. In this study, α is set at 0.05. Therefore, Z_α/2_ or Z_0.025_ corresponds to 1.96 for a 95% confidence interval. V denotes the coefficient of variation, Δ is an acceptable difference between the true population mean and the sample estimate, and D is the design effect. The values for V, D, and Δ in this study were set at 2, 1.5, and 0.15, respectively, in accordance with the study on evaluating the willingness to pay in Thailand’s context by Thavorncharoensap et al.^23^ As a result, the required sample size is 1025. To account for the incomplete interviews, the sample size was adjusted to 1200 subjects.

The following subjects were eligible for this study: (1) Chiang Mai Province residents aged 25–50 years (as of September 30th, 2020) with Thai nationality according to the civil registration; (2) the participants must have lived in their own residence for at least 1 year before the data collection date; (3) be able to read and write Thai; and (4) be able to provide informed consent. The subjects who presented with a disability, acute illness, or cognitive impairment, or who had been unemployed for more than 1 year before taking the questionnaire were excluded.

A stratified multistage random sampling technique was performed to recruit the subjects in Chiang Mai Province, Thailand. To begin, all districts in Chiang Mai Province were classified into 5 groups based on their geographic locations and the Chiang Mai Province’s patient referral system. Next, a quota sampling technique was used to select the districts based on the population ratios among groups. All districts in groups 1 and 2 were selected. For groups 3–5, two districts per group were randomly selected. As a result, 16 out of 25 districts in Chiang Mai Province were selected. For each selected district, 2–4 subdistricts were purposely selected. The accidental sampling method was used to select subjects from the general Thai population who met the eligibility criteria. Required and actual samples for each selected district are presented in Table S1.

### Procedure

A total of 1202 subjects were asked to complete the money-related questionnaire. For health-related questions, all subjects were randomly assigned in a 1:1 ratio. A total of 602 subjects were assigned to the first group and were asked to complete a questionnaire about the COVID-19 situation. For the second group, 600 people completed a questionnaire about scenarios of air pollution with fine particulate matter.

### Study instrument

The questionnaires were developed based on the literature review and were revised in response to recommendations from the expert meeting. There were two versions of the questionnaire. The first version was associated with COVID-19 scenarios, whereas the second version was involved with air pollution caused by fine particulate matter, or PM2.5. Each version included the following 3 major parts: (1) socio-demographic data; (2) health status; and (3) time preferences for money and health. The questionnaires were tested for content validity based on the expert meeting’s recommendations. Following the development of all questionnaires, a face validity test was conducted.

The experiment was run on a web-based computer and was developed based on a questionnaire involving time preferences for money and health (part 3 in each of the questionnaires). The web-based experiment was also tested for validity and reliability.

### Outcomes

The primary outcome of this study was the discount rates for money and health determined from the subjects' indifference values and the secondary outcome was the socio-demographic characteristics that influence discounting.

### Data collection

The officers in local organizations such as the District Office, District Public Health Office, Sub-district Municipality, and Health Promoting Hospital were contacted to coordinate and recruit the volunteers or subjects in the selected districts. The general Thai population that met the eligibility criteria of this study was included in the study. The questionnaire survey sessions were conducted in local government meeting rooms near the study subjects’ homes. Depending on the number of subjects, there were 12–15 respondents per session and 4–6 sessions per day. After respondents registered, they were assigned a well-trained interviewer to help them with the interview. All interviewers were trained to standardize the data collection process, including the study protocol, informed consent, and experimental design, and they also underwent a practice with a pilot sample.

Face-to-face, computer-assisted, one-on-one interviews were performed to collect data. The questionnaires consist of 3 parts: (1) demographic data, (2) health data, and (3) time preference experiment. The lead instructor explained each step of this survey, but the standardized details were presented to the respondents via the instructional video clips. Subjects privately completed the demographic data and answered the health questions with the help of the interviewer. In the web-based experiment, subjects decided and indicated their preferred option in Fig. 1 (option A or B). The interviewers then assisted in entering the selected option to avoid errors. The time required for the entire process was estimated to be 40–60 min per session. Data collection lasted approximately 4 months, from April to July 2021.

### Data analyses

The statistical analyses were divided into 4 steps. First, a choice-based elicitation procedure was performed to measure the individual’s indifference values of time preference for money and health outcomes. Second, the indifference values from each subject were summarized using descriptive statistics. Third, the cumulative weighting functions were generated using the indifference values from each time point and the discount rates were measured using a continuous approximation. Finally, the socio-demographic variables influencing the discount rate were explored. The detailed information was provided as follows.

#### Step 1: measurement of individual indifference values

A choice-based elicitation procedure was performed to obtain the subjects’ time preferences for money and health. The health and money profiles covered the next 20-year time interval. The time interval was divided into 7 time points: t_0_, t_.125_, t_.25_, t_.5_, t_.75_, t_.875_, and t_1_, respectively. The subjects’ current age was represented by time point t_0_, and their age in the next 20 years was represented by time point t_1_. The elicitation was performed in 4 iterations at each time point.

The elicitation procedure began at time point t_.5_. In the first iteration, each subject was asked to choose between two options (A vs. B) for their preferred period. For the 2nd and 3rd iterations, the period in the selected option was shortened by half to make the selected option less attractive and the non-chosen option more attractive. After three iterations, the cut-off value for the 4th iteration was calculated as the indifference value by averaging the smallest value of the preferred option A and the largest value of the preferred option B. After 4 iterations, the indifference value of time point t_.5_ was analyzed to determine the earlier benefit for which a subject was indifferent concerning a given future benefit. Table S2 reveals an example of choice-based elicitation for time point t_.5_.

Next, the elicitation of the remaining 4 time points was examined. The order of elicitation at time points t_.25_ and t_.75_ was then assigned at random. After that, the order of elicitation at time points t_.125_ and t_.875_ was randomized. Finally, the indifference values for 5 time points per subject were collected.

Because the remaining intervals were narrowed to allow eliciting new values, some subjects did not complete all questions. The subjects who always selected all first-period options or all later-period options were categorized as extremely impatient or extremely patient, respectively. Those subjects who preferred the first-period option (t_.125_ = t_.25_ = 0) were labeled as impatient with those preferring the later-period option (t_.0.75_ = t_.875_ = 20) as patient. These four types of subjects were grouped as ‘extreme subjects.’ The remainders, on the other hand, were classified as ‘non-extreme subjects.’

The subjects with extreme preferences were excluded from the measurements of cumulative weighting functions and discount rates. However, all subjects were included in the analysis of the relationships between discounting and socio-demographic characteristics using the Tobit model.

#### Step 2: summary of the indifference values

The descriptive statistics were used to summarize the indifference values from the choice-based elicitation and were displayed as mean, standard deviation (SD), median, and interquartile range (IQR).

#### Step 3: measurement of cumulative weights

The cumulative weighting functions were generated using the mean and median of the indifference values from each time point. The degree of discounting was indicated by the area under the cumulative weighting function. The greater the size of this area, the more the subjects discount the future.

#### Step 4: measurement of discount factors and discount rates

The discount factors were computed from the cumulative weighting functions in the discounting model^17^. Due to only 5 data points per subject being obtained, the following one-parameter discounting models were used: (1) constant discounting; (2) proportional discounting; (3) power discounting; (4) dual exponential discounting; and (5) periodic discounting model. The cumulative weighting functions and discount factor equations for each discounting model are listed in Table 1.

**Table 1:** Cumulative weighting functions and discount factor equations.

Discounting model	Cumulative weighting function $${C}_{({t}_{j})}$$	Discount factor ($${d}_{t})$$
(1) Constant discounting	${C}_{({t}_{j})}= \frac{1-{e}^{-\delta {t}_{j}}}{1-{e}^{-{\delta }_{T}}}$	${d}_{t}=\frac{1}{1+\delta }$
(2) Proportional discounting	${C}_{({t}_{j})}= \frac{\mathrm{ln}(1+\kappa {t}_{j})}{\mathrm{ln}(1+\kappa T)}$	${d}_{t}=\frac{1}{1+\kappa t}$
(3) Power discounting	${C}_{({t}_{j})}= \frac{{(1+{t}_{j})}^{1-\propto }-1}{{(1+T)}^{1-\propto }-1}$	${d}_{t}={\left(\frac{1}{1+t}\right)}^{\propto }$
(4) Dual exponential discounting	${C}_{({t}_{j})}= \frac{{{t}_{j}-e}^{r{t}_{j}}-{e}^{-r{t}_{j}}}{{T-e}^{rT}-{e}^{-rT}}$	${d}_{t}=0.5{e}^{-rt}-0.5{e}^{rt}+1$
(5) Periodic discounting	${C}_{({t}_{j})}= \frac{{\rho t}_{j}-\mathrm{sin}\left({\rho t}_{j}\right)+(\mathrm{cos}\left({\rho t}_{j}\right)-1)}{\rho T-\mathrm{sin}\left(\rho T\right)+(\mathrm{cos}\left(\rho T\right)-1)}$	${d}_{t}=0.5+0.5\mathrm{cos}\left(\rho t\right)-0.5\mathrm{sin}(\rho t)$

Remark: δ, κ, ∝ , r, and p represent parameters governing discounting model.

To determine which discount model best described subjects' preferences, this study used these 5 discounting models to fit the cumulative weighting functions. The subject’s indifference values for 5 time points were used to compute the individual parameters for 5 discounting models. The squared error for each model is calculated using the following equation.

$$\mathop \sum \limits_{j = 1}^{5} \left( {C\left( {t_{j} } \right) - C\left( {\hat{t}_{j} } \right)} \right)^{2} \;{\text{where}}\;C\left( {\hat{t}_{j} } \right) = \left[ {0.125,\;0.25,\;0.5,\;0.75,\;0.875} \right].$$

Then, the individual best-fitting model was selected based on the minimum squared error. Finally, the best-fitting discounting model was the one with the highest proportion of subjects for whom each of the discount models fit best.

Discount rates, or the rates at which future consequences are devalued, were estimated using a continuous approximation based on the relationship between the discount factors and the parameter governing the discounting model. Each discounting model yielded the discount rate results. The discount rate results were chosen using the best-fit discounting model.

### The effect of socio-demographic variables on discounting

Because the areas under the normalized weighting functions were censored between 0 and 1, the Tobit model was applied to investigate the relationships between discounting and socio-demographic characteristics^24^. There were 5 different models: model I combined discounting for money and health to test whether discounting was domain-specific; models II and III separately regressed discounting for money and health; models IV and V regressed health discounting in the COVID-19 and air pollution situations, respectively.

### Data analysis tools

Descriptive statistics and the Tobit model were performed using the STATA software version 14.0 (StataCorp. 2015. Stata Statistical Software: Release 14. College Station, TX: StataCorp LP). The continuous approximation for discount rate was analyzed using R software (R Core Team. 2013. R: A language and environment for statistical computing. R Foundation for Statistical Computing, Vienna, Austria).

### Ethical approval and consent to participate

The ethics approval was obtained from the Institutional Review Board of the Faculty of Pharmacy, Chiang Mai University (Certificate of Approval No.006/2564/E). All procedures were carried out in accordance with the applicable guidelines and regulations. The study protocol was explained to all subjects. All subjects signed informed consent forms after agreeing to participate in the study.

## Results

### Demographic characteristics

A total of 1202 subjects participated in the experiment, ranging from 25 to 50 years old, with a mean age of 37.3 ± 7.9 years. Of those, 66.6% were female, 40.9% were generation X (aged 42 years and above), and 42.4% were married. The average number of years of education was 12.9 (standard deviation; SD 0.1). The average monthly household income was 30,497 THB (SD 41,611). In the money and health scenarios, the extreme subjects were 47.9% and 62.5%, respectively. Except for underlying diseases that are risk factors for COVID-19 and/or respiratory diseases, all characteristics were similar between COVID-19 and air pollution situations. The detailed descriptions of the subjects’ characteristics are reported in Table 2.

**Table 2:** Socio-demographic characteristics.

Characteristics	Number of subjects (Percent)			
	COVID-19 (N = 602)	Air pollution (N = 600)	Total (N = 1202)	*p*-value*
(1) Male	200 (33.2)	201 (33.5)	401 (33.4)	0.918
(2) Mean age (standard deviation)	37.3 (7.9)	37.3 (7.9)	37.3 (7.9)	0.925
(3) Generation X subjects	255 (42.4)	237 (39.5)	492 (40.9)	0.313
(4) Married	260 (43.2)	249 (41.5)	509 (42.4)	0.553
5) Children below 15 years of age in the household	292 (48.5)	309 (51.5)	601 (50.0)	0.299
(6) Elderly in the household (aged ≥ 60 years)	301 (50.0)	276 (46.0)	577 (48.0)	0.165
(7) Mean years of education (standard deviation)	13.1 (0.1)	12.8 (0.2)	12.9 (0.1)	0.126
(8) Government sector career	224 (37.2)	225 (37.5)	449 (37.4)	0.917
(9) Underlying diseases that are risk factors for COVID-19 and/or respiratory diseases	149 (24.8)	58 (9.7)	207 (17.2)	< 0.001
(10) Current smoker	75 (12.5)	88 (14.7)	163 (13.6)	0.263
(11) Mean household income per month (standard deviation)	29,242 (25,843)	31,757 (52,990)	30,497 (41,611)	0.294
(12) Extreme subjects				
Money	287 (47.6)	289 (48.2)	576 (47.9)	0.865
Health	389 (64.6)	362 (60.3)	751 (62.5)	0.125
(13) Money-related scenario first presented to subjects	305 (50.7)	306 (51.0)	611 (50.8)	0.907

Remark: * Statistical analysis using independent t-test.

### Cumulative weighting functions

**Figure 2:**
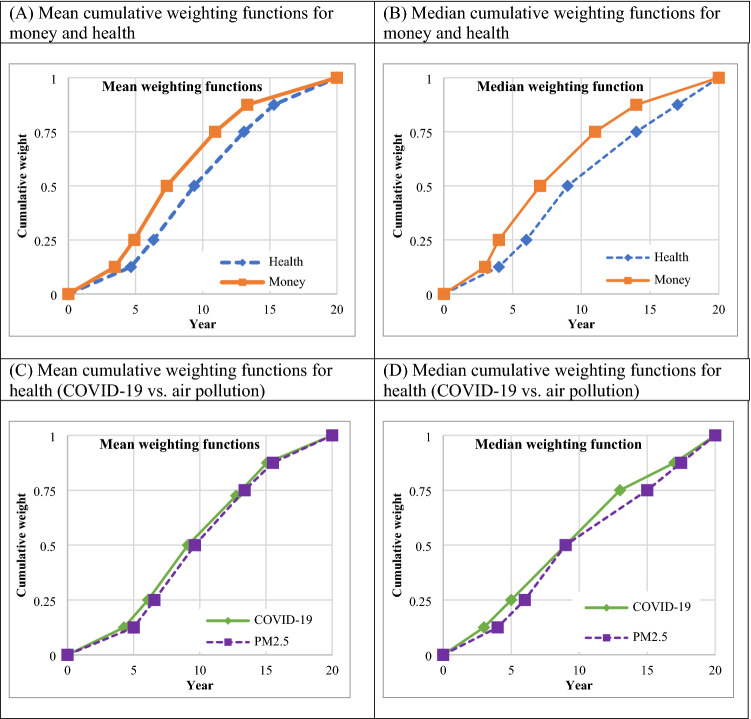
Cumulative weighting functions. COVID-19, Coronavirus disease 2019; PM2.5, Air pollution with fine particulate matter.

A table with descriptive statistics was presented in the Supplement (Table S3). Statistical analyses confirmed that the mean indifference values for health were significantly higher than the mean indifference values for money at all time points (Wilcoxon test, p < 0.001). The median and mean cumulative weighting functions for money and health are demonstrated in Fig. 2. The cumulative weighting function for money was greater than that for health, as shown in Fig. 2A and B. Therefore, this indicates that there was more discounting for money than for health. The degree of discounting is indicated by the cumulative weighting function. The mean areas for money and health were 0.592 and 0.513, respectively, while the median areas for money and health were 0.603 and 0.506, indicating a positive discounting. Statistical tests revealed that the elicited values differed significantly between money and health (ANOVA with repeated measures, p < 0.001).

The mean areas for the COVID-19 situation and the air pollution situation in the health scenario (Fig. 2C) were 0.523 and 0.502, respectively, while the median areas were 0.531 and 0.494. These results indicated that the COVID-19 situation was discounted more than the air pollution situation. However, the values elicited were similar in both situations (ANOVA with repeated measures, p 0.899).

**Figure 3:**
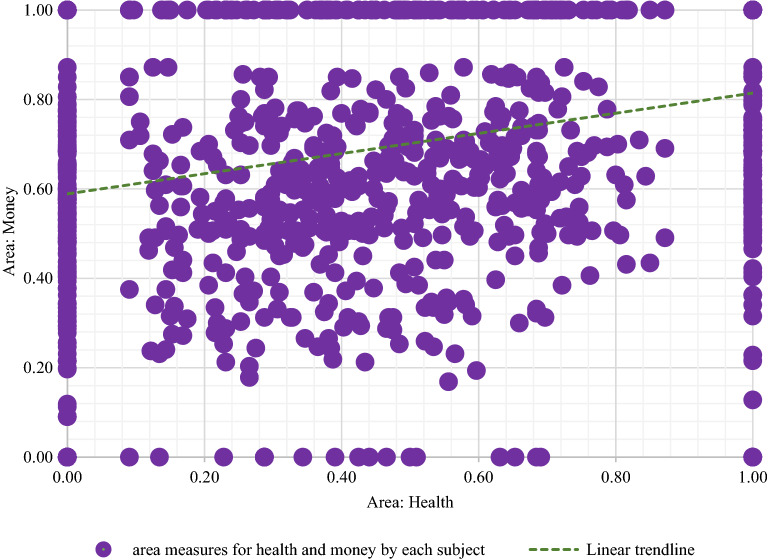
Relation between the area measures for money and health.

Figure 3 depicts the relation between the health and the money area measures. The number of data points was reflected in the dots. The correlation between discounting for health and discounting for money was moderate (Kendall’s correlation coefficient 0.202). The positive correlation between non-monetary outcome and monetary outcome founded in this study supported the delay discounting^8^.

### Discounting models

The analyses used 5 parametric forms to fit the cumulative weighting functions to determine which discount function best described subjects’ preferences. The medians of the individual estimates of the parameters in each of the models are shown in Table 3. In addition, constant discounting provided the best fit for 46.49% of the health subjects and 59.75% of the money subjects (Table S4).

**Table 3:** Estimated discount functions.

Scenario	Estimated discount functions (median, [quantile])				
	Constant discounting model	Proportional discounting model	Power discounting model	Dual exponential discounting model	Periodic discounting model
Health	0.013 [− 0.053, 0.096]	0.149 [− 0.031, 0.271]	0.115 [− 0.561, 0.659]	0.090 [0.000, 0.120]	0.530 [0.230, 1.250]
Money	0.062 [0.011, 0.137]	0.122 [0.135, 0.577]	0.463 [0.104, 0.862]	0.010 [0.070, 0.120]	0.445 [0.190, 1.050]
COVID-Health	0.024 [− 0.043, 0.117]	0.032 [− 0.028, 0.359]	0.203 [− 0.442, 0.722]	0.090 [0.000, 0.120]	0.570 [0.230, 1.100]
COVID-Money	0.068 [0.015, 0.141	0.143 [0.017, 0.610]	0.511 [0.131, 0.871]	0.100 [0.080, 0.120]	0.470 [0.200, 1.115]
PM-Health	0.007 [0.001, 0.085]	0.007 [0.006, 0.212]	0.061 [− 0.593, 0.593]	0.090 [0.000, 0.110]	0.210 [− 0.480, 0.517]
PM-Money	0.058 [0.006, 0.130]	0.109 [0.007, 0.514]	0.438 [0.057, 0.831]	0.114 [0.080, 0.129]	0.467 [0.220, 1.270]

Remarks: (1) Estimated discount functions based on non-extreme subjects.

(2) [ ] depicts 1st and 3rd quantile of discount factor.

According to the estimated parameters in the constant discounting model (Table 3), the discount rate for money was higher than the discount rate for health, at 6.2% and 1.3%, respectively. Meanwhile, the discount rate for health in the COVID-19 situation was higher than that in the air pollution situation, at 2.4% and 0.7%, respectively.

**Figure 4:**
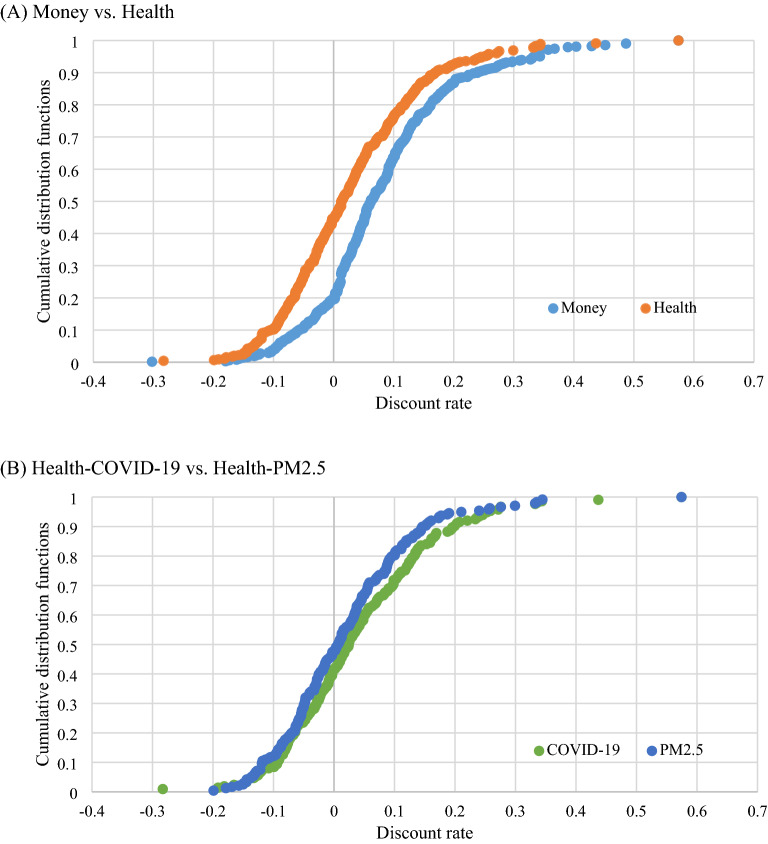
Cumulative distribution functions of the discount rates. Representations: COVID-19 = Coronavirus disease 2019, PM2.5 = Air pollution with fine particulate matter.

Figure 4 demonstrates the cumulative distribution functions of the discount rates for money and health. The distribution for health was mostly above the distribution for money, indicating that money was discounted more than health (Fig. 4A). Furthermore, the distribution for the air pollution situation was mostly higher than that for the COVID-19 situation, indicating that the COVID-19 situation was discounted more than the air pollution situation (Fig. 4B).

### Effect of socio-demographic characteristics on discounting

Table 4 shows the estimation results of the Tobit models that described the area under the normalized cumulative weighting functions for money and health by socio-demographic variables. The pseudo-R^2^ in each model indicates that the goodness of fit is low.

**Table 4:** The effect of socio-demographic characteristics on discounting.

Characteristics	The area under the normalized utility function				
	All samples	Money	Health	Health (COVID-19)	Health (air pollution)
Male	− 0.033	− 0.021	− 0.044	0.004	− 0.089
	(0.028)	(0.034)	(0.046)	(0.073)	(0.058)
Generation X	0.117***	0.023	0.223***	0.223***	0.200***
	(0.027)	(0.032)	(0.043)	(0.070)	(0.053)
Married	0.012	0.020	0.005	− 0.017	0.018
	(0.027)	(0.032)	(0.044)	(0.070)	(0.054)
Children below 15 years of age in the household	0.056**	0.044	0.069*	0.160**	− 0.006
	(0.025)	(0.030)	(0.041)	(0.066)	(0.051)
Elderly in the household (aged ≥ 60 years)	− 0.020	− 0.014	− 0.026	− 0.031	− 0.019
	(0.024)	(0.028)	(0.038)	(0.062)	(0.047)
Years of education	− 0.005	0.0003	− 0.010	− 0.011	− 0.006
	(0.004)	(0.005)	(0.007)	(0.012)	(0.008)
Government sector career	− 0.006	0.008	− 0.023	− 0.065	0.005
	(0.029)	(0.035)	(0.047)	(0.077)	(0.058)
Underlying diseases that are risk factors for COVID-19 and/or respiratory diseases	0.067**	0.036	0.099*	0.168**	− 0.030
	(0.032)	(0.038)	(0.052)	(0.074)	(0.079)
Current smoker	− 0.007	− 0.060	0.053	0.052	0.061
	(0.040)	(0.047)	(0.064)	(0.105)	(0.078)
Household income	− 0.061***	− 0.047**	− 0.078***	− 0.073	− 0.088***
	(0.018)	(0.021)	(0.028)	(0.048)	(0.034)
Money-related scenario first	− 0.027	− 0.087***	0.046	0.009	0.078*
	(0.024)	(0.028)	(0.038)	(0.061)	(0.047)
COVID-19 situation	0.101***	0.029	0.184***		
	(0.024)	(0.029)	(0.039)		
Money scenario	0.379***				
	(0.024)				
Constant	1.002***	1.259***	1.102***	1.223**	1.202***
	(0.166)	(0.198)	(0.268)	(0.448)	(0.319)
Sigma	0.540***	0.456***	0.615***	0.695***	0.539***
	(0.012)	(0.014)	(0.020)	(0.034)	(0.020)
pseudo-R^2^	0.076	0.014	0.046	0.042	0.038
Number of datasets	2404	1202	1202	602	600

COVID-19, Coronavirus Disease 2019.

Remarks: (1) ( ) depicts the standard error.

(2) Tobit model, with the left-censored value at 0 and the right-censored value at 1.

(3) *,**,*** denote statistically significant at the 0.01, 0.05, and 0.1 levels, respectively.

Overall (Model I), several socio-demographic characteristics including generation X subjects (aged 42 years and above), children under the age of 15 in the household, and underlying diseases that are risk factors for COVID-19 and/or respiratory diseases, are positively related to discounting, while household income is negatively related. However, other variables such as marital status, education, occupation, and smoking had no effect on the discounting rate. The pooled regression reveals that discounting for money differed from discounting for health (p 0.024), confirming that discounting was domain-specific in our study.

Household income and money-related scenario first presented to subjects were negatively related to discounting in Model II (money scenario). Being generation X subjects was positively related to discounting in Models III (health scenario), IV (COVID-19 situation), and V (air pollution situation). Moreover, in Model III (health scenario) and Model IV, the COVID-19 situation, children under the age of 15 in the household, and underlying diseases that are risk factors for COVID-19 and/or respiratory diseases were positively related to discounting, while household income was negatively related to discounting in Models III and V.

## Discussion

To the best of our knowledge, this study is the experimental attempt to use a direct method to obtain time preferences for money and health in a large representative sample in Thailand. As the subjects discounted money more than health, discounting in this study was domain-specific. The correlation between discounting for money and health was moderate (Kendall’s correlation coefficient 0.202). These findings are in line with previous studies^17,25^. In particular, the study conducted by Attema et al.^17^ reported Kendall's correlation coefficient of 0.22. As a result, we found the annual discount rate for money to be higher than that for health, at 6.2% and 1.3%, respectively. Furthermore, the annual discount rate for health in the COVID-19 situation was higher than that in the case of air pollution, at 2.4% and 0.7%, respectively.

Our findings regarding the discount rates are consistent with those of a previous study conducted by Attema et al.^17^. Their experiment was carried out on subjects aged 30–50 years, which is comparable to our study. The annual discount rates for money and health estimated from their study were 6.5% and 2.2%, respectively. However, these results differ from the findings from a study conducted by Fredslund et al.^18^ which reveals the annual discount rate for money to be lower than that for health, at 27.8% and 29.4%, respectively. In contrast to our study, their subjects ranged in age from working to retirement age.

Furthermore, from the present study, the discount rate for health in the COVID -19 situation was higher than that in the air pollution situation (2.4% vs. 0.7%). This could be because the health consequences are different between communicable diseases and noncommunicable diseases; thus, leading to differences in risk perception and discounting degree. COVID-19 is the current pandemic that would have a significant impact on the population and economy at both the individual and the societal levels. As a result, there is an urgent need to protect against COVID-19 infection rather than the long-term effects caused by air pollution including respiratory diseases.

Furthermore, the pooled regression reveals that discounting for money differed from discounting for health (p 0.024), confirming that the discounting was domain-specific. A statistically significant discount was found to be positively correlated with the sample data in the money situation. This means that discounting for money is higher than discounting for health. According to Attema et al.^17^, the subjects who answered questions about money were positively correlated with a statistically significant discount. This is a piece of empirical evidence to confirm that discounting money and health are not the same thing. Health should be discounted at a lower rate than money.

Several limitations in this study should be taken into consideration. First, the study gathered data on time preferences for money and health in a large representative sample of the Chiang Mai Province population. This may limit the study’s generalizability to other provinces or Thailand's representatives. However, the findings of this study might be useful for future studies into determining appropriate discount rates for money and health in the context of health economic evaluation in Thailand. Second, COVID-19 and air pollution situations were used in this study to reflect the current health issues in Chiang Mai Province, particularly, the air pollution with fine particulate matter that occurs every year from January to April has been a common concern. As a result, these situations may have limited generalizability once applied to other contexts. Third, most subjects examined had extreme preferences. This might be due to the data were gathered between April and July 2021, which was the period of the COVID-19 pandemic in Thailand. There were limited vaccinations available to Thais at the time. COVID-19 had an impact on the economy at both the individual and the societal levels. Furthermore, air pollution from PM2.5 was a significant health concern in northern Thailand, particularly in Chiang Mai province. Therefore, there was an urgent need of the given benefits from both money and health scenarios. However, we established the study protocol and trained all interviewers to standardize the data collection process and avoid errors. Each step of the experimental design was explained by the interviewer. The respondents were shown the standardized information via instructional video clips. Before the experiment began, the subjects had the opportunity to request additional information or clarification.

## Conclusion

This study’s findings revealed that discounting for money was greater than discounting for health. Money and health had annual discount rates of 6.2% and 1.3%, respectively. Furthermore, in the COVID -19 situation, the annual discount rate for health was higher than that in the air pollution situation (2.4% vs. 0.7%). Health should be discounted at a lower rate than money. In addition, different discount rates should be considered for different types of diseases.

## Supplementary Information

Supplementary Tables.

## Data Availability

All data generated or analyzed during this study are included in this published article and its supplementary information file. Additional raw data files can be available from the corresponding author upon reasonable request.
