# Supplementary material for: Discounting money and health effects from communicable and noncommunicable diseases in Thailand
Source: Sci Rep. 2023 Feb 27;13:3324. doi: 10.1038/s41598-023-30559-2 (PMC9969024; doi:10.1038/s41598-023-30559-2)
Supplement: Supplementary file 1 — Supplementary Tables. [file 41598_2023_30559_MOESM1_ESM.pdf]

Supplement data

Table S1 Population and sample sizes

Group	District	Number of population* (persons)	Percentage of total population (%)		Number of required samples by group (subjects)	Number of required samples by selected districts (subjects)	Number of actual samples (subjects)		
			by district	by group			Scenario: COVID-19	Scenario: Air pollution	Total
1	Mueang Chiang Mai	222,116	13.7	13.7	165	165	86	73	159
2	San Sai	131,892	8.2	42.0	504	98	49	48	97
	Hang Dong	93,036	5.8			69	31	39	70
	San Kamphaeng	86,263	5.3			64	36	29	65
	Mae Rim	85,460	5.3			63	29	34	63
	Saraphi	83,546	5.2			62	23	45	68
	San Pa Tong	74,081	4.6			55	27	28	55
	Doi Saket	72,749	4.5			54	29	24	53
	Mae Wang	31,375	1.9			23	14	9	23
	Mae On	20,931	1.3			16	8	9	17
3	Fang	89,659	5.5	12.0	144	101	52	50	102
	Mae Ai	66,180	4.1			-	-	-	-
	Chai Prakan	38,468	2.4			43	28	15	43
4	Chiang Dao	72,488	4.5	15.2	182	92	52	40	92
	Mae Taeng	71,457	4.4			90	36	54	90
	Pharo	47,843	3.0			-	-	-	-
	Samoeng	23,038	1.4			-	-	-	-
	Wiang Haeng	18,597	1.1			-	-	-	-
	Galyani Vadhana	12,556	0.8			-	-	-	-
5	Chom Thong	65,799	4.1	17.1	205	-	-	-	-
	Omkoi	61,110	3.8			127	62	65	127
	Mae Chaem	59,309	3.7			-	-	-	-
	Hot	37,556	2.3			78	40	38	78
	Doi Tao	27,281	1.7			-	-	-	-
	Doi Lo	25,046	1.5			-	-	-	-
Total		1,617,836	100	100	1,200	1,200	602	600	1,202

* Data were obtained from the Bureau of Registration Administration, Department of Provincial Administration, Ministry of Interior, Thailand (data as of December 31st, 2019).

Table S2 Choice-based elicitation for time point $t_{.5}$: an example for a 30-year-old subject

Iteration	Question: which do you prefer? To be healthy from the benefit of vaccination at age		Answer	Indifference value
	**Option A**	**Option B**		
1	30-**40**	40-50	A	-
2	30-35	**35**-50	B	-
3	30-**43**	43-50	A	38[*]
4	30-**38**	38-50	A	37[#]

[*] The smallest value of the preferred option A and the largest value of the preferred option B are 40 and 35, respectively.

[#] The smallest value of the preferred option A and the largest value of the preferred option B are 38 and 35, respectively,

A choice-based elicitation procedure was performed to obtain the subjects' time preferences for money and health. The health and money profiles covered the next 20-year time interval.

The 1st iteration

In each time point, the elicitation was performed in 4 iterations. At the first iteration of $t_{.5}$ elicitation, the 20-year interval was cut in half to make the option indifferent in terms of time interval. Therefore, the periods of options A and B equal 10 years. According to Table S2, a 30-year-old subject was asked to choose the preferred period to be healthy from the benefit of COVID-19 vaccination.

The 2nd iteration

According to the selected option A in the first iteration. The period in the selected option was shortened by half to make the selected option less attractive and the non-chosen option more attractive. Therefore, the periods of option A and B are at age 30-35 and 35-50 years. The subject was asked to choose a preferred period from the new options.

The 3rd iteration

The subject selected option B in the 2nd iteration. The period in the selected option was shortened by half. As a result, the periods of option A and B for the 3rd iteration are at age 30-43 and 43-50 years. The subjects chose option A.

After 3 iterations, the cut-off value for the 4th iteration was calculated as the indifference value by averaging the smallest values of the preferred option A and the largest values of the preferred option B. The smallest value of option A and the largest value of option B are 40 and 35, respectively. Therefore, the calculated indifference value after three iterations is 38, which was used as the cut-off value for the 4th iteration.

The 4th iteration

According to the calculated indifference value after three iterations, the periods of option A and B for the 4th iteration are at age 30-38 and 38-50 years. The subjects chose option A in this iteration.

After 4 iterations, the indifference value of time point $t_{.5}$ was analyzed to determine the earlier benefit for which a subject was indifferent with regard to a given future benefit. The smallest value of the preferred option A and the largest value of the preferred option B are 38 and 35. As a result, the indifference value of time point $t_{.5}$ was calculated to be 37 after four iterations.

Table S3 Summary of indifference values

	Indifference values				
	$t_{0.125}$	$t_{0.25}$	$t_{0.5}$	$t_{0.75}$	$t_{0.875}$
**1) Money (N=626)**					
Mean (SD)	3.46 (3.09)	4.91 (3.29)	7.33 (3.89)	10.93 (4.56)	13.32 (5.05)
Median (IQR)	3 (4)	4 (4)	7 (4)	11 (8)	14 (9)
**2) Health (N=451)**					
Mean (SD)	4.66 (3.89)	6.34 (3.95)	9.37 (4.31)	13.08 (4.60)	15.31 (4.65)
Median (IQR)	4 (5)	6 (6)	9 (7)	14 (8)	17 (6)
2.1 COVID-19 (N=213)					
Mean (SD)	4.27 (4.02)	6.08 (4.08)	9.09 (4.28)	12.74 (4.61)	15.08 (4.71)
Median (IQR)	3 (5)	5 (7)	9 (7)	13 (8)	17 (8)
2.2 Air pollution (N=238)					
Mean (SD)	5.00 (3.75)	6.57 (3.82)	9.62 (4.33)	13.38 (4.58)	15.52 (4.60)
Median (IQR)	4 (5)	6 (5)	9 (7)	15 (7)	17.5 (6)
**Wilcoxon tests (p-value)**					
Money vs. Health	<0.001	<0.001	<0.001	<0.001	<0.001
COVID-19 vs. Air pollution	0.003	0.094	0.194	0.119	0.356
**Analysis of Variance with repeated measure (F-stat, p-value)**					
Money vs. Health	F = 7.85***		0.0000		
COVID-19 vs. Air pollution	F = 0.27		0.8994		

Abbreviation: COVID-19, Coronavirus Disease 2019; IQR, interquartile range; SD, standard deviation

Remark: Data were obtained from non-extreme subjects.

Table S4 Proportion of subjects for whom each of the discounting models fitted best

Scenario	Proportion of subjects for whom each of the discounting models fitted best (Percent)				
	Constant discounting model	Proportional discounting model	Power discounting model	Dual exponential discounting model	Periodic discounting model
Health	**46.49%**	6.66%	22.61%	6.17%	18.07%
Money	**59.75%**	12.22%	19.36%	3.21%	5.46%
COVID-19-Health	**36.01%**	13.00%	13.66%	5.56%	31.78%
COVID-19-Money	**67.90%**	11.30%	13.50%	3.20%	4.20%
PM-Health	**36.12%**	16.09%	17.28%	15.37%	15.14%
PM-Money	**30.47%**	11.03%	30.08%	13.57%	14.85%

Abbreviation: COVID-19, Coronavirus Disease 2019

Remarks: 1) Data were obtained from non-extreme subjects.

2) Bold indicates the best fit model.
